# Herbivory and dominance shifts among exotic and congeneric native plant species during plant community establishment

**DOI:** 10.1007/s00442-015-3472-6

**Published:** 2015-10-19

**Authors:** Tim Engelkes, Annelein Meisner, Elly Morriën, Olga Kostenko, Wim H. Van der Putten, Mirka Macel

**Affiliations:** Department of Terrestrial Ecology, Netherlands Institute of Ecology (NIOO-KNAW), P.O. Box 50, 6700 AB Wageningen, The Netherlands; Laboratory of Nematology, Wageningen University and Research Centre, P.O. Box 8123, 6700 ES Wageningen, The Netherlands; Microbial Ecology Group, Department of Biology, Lund University, Ecology Building, 223 62 Lund, Sweden; Sections of Microbiology and Terrestrial Ecology, Department of Biology & Center for Macroecology, Evolution, and Climate, The Natural History Museum of Denmark, University of Copenhagen, Universitetsparken 15, 2100 København Ø, Denmark; Department of Plant Ecology, University of Tuebingen, Auf der Morgenstelle 5, 72076 Tuebingen, Germany

**Keywords:** Biological invasion, Phylogenetically controlled experiment, Community evenness, Biotic interactions, Enemy release

## Abstract

Invasive exotic plant species often have fewer natural enemies and suffer less damage from herbivores in their new range than genetically or functionally related species that are native to that area. Although we might expect that having fewer enemies would promote the invasiveness of the introduced exotic plant species due to reduced enemy exposure, few studies have actually analyzed the ecological consequences of this situation in the field. Here, we examined how exposure to aboveground herbivores influences shifts in dominance among exotic and phylogenetically related native plant species in a riparian ecosystem during early establishment of invaded communities. We planted ten plant communities each consisting of three individuals of each of six exotic plant species as well as six phylogenetically related natives. Exotic plant species were selected based on a rapid recent increase in regional abundance, the presence of a congeneric native species, and their co-occurrence in the riparian ecosystem. All plant communities were covered by tents with insect mesh. Five tents were open on the leeward side to allow herbivory. The other five tents were completely closed in order to exclude insects and vertebrates. Herbivory reduced aboveground biomass by half and influenced which of the plant species dominated the establishing communities. Exposure to herbivory did not reduce the total biomass of natives more than that of exotics, so aboveground herbivory did not selectively enhance exotics during this early stage of plant community development. Effects of herbivores on plant biomass depended on plant species or genus but not on plant status (i.e., exotic vs native). Thus, aboveground herbivory did not promote the dominance of exotic plant species during early establishment of the phylogenetically balanced plant communities.

**Electronic supplementary material:**

The online version of this article (doi:10.1007/s00442-015-3472-6) contains supplementary material, which is available to authorized users.

## Introduction

Exotic plant species can pose a major threat to the biodiversity and functioning of ecosystems worldwide, because some exotics change the cycling of carbon, nutrients, and water, as well as interactions with other plants and animals (Lodge 1993; Mack et al. 2000; Richardson et al. 2000). During invasion, exotic plant species pass through several bottlenecks, including the introduction of propagules, colonization, establishment, and spread (Colautti et al. 2004; Theoharides and Dukes 2007; Catford et al. 2009). Biotic interactions with herbivores and other enemies may influence the performance of an exotic plant species during each of these phases, either counteracting or promoting invasion (Keane and Crawley 2002; Parker and Hay 2005). Although many studies have focused on effects of herbivory on established invasions, relatively few empirical studies have aimed at examining how herbivory may influence plant dominance during plant community establishment in the field (Newingham and Callaway 2006; Gonzales and Arcese 2008). Examining this phase of community development is an important task because many plant invasions occur in disturbed habitats. Here, we report the results of a field study in which establishing plant communities in a riparian ecosystem were either exposed to herbivory or not. Riparian ecosystems are disturbed periodically by winter floods, necessitating regular plant community re-establishment.

Many studies have suggested that exotic species in their new range are less affected by natural enemies than species native to that area (Keane and Crawley 2002; Mitchell and Power 2003; Mitchell 2010). Indeed, field surveys generally indicate that introduced exotics have fewer enemies than natives (Mitchell and Power 2003; Torchin et al. 2003), or fewer specialized enemies (Jobin et al. 1996; Memmott et al. 2000). However, not all field survey studies support this view. For example, the geographical spread of North American exotic plant species in Europe could not be fully related to their release from floral and foliar fungal pathogens (Van Kleunen and Fischer 2009). Moreover, field tests on how enemy exposure may affect invasiveness in plant communities are relatively rare (Levine et al. 2003; Liu and Stiling 2006) and show variable effects of enemy exposure on invasion (Stohlgren et al. 1999; Holmgren et al. 2000; Wolfe et al. 2004; Agrawal et al. 2005; Parker and Hay 2005; Parker and Gilbert 2007; Catford et al. 2009; Dawson et al. 2014).

Effects of herbivores on exotic plant species in the field are often influenced by a variety of factors. For example, the impact of herbivory on exotic species can depend on the composition of the surrounding vegetation (Prieur-Richard et al. 2002). Effects of herbivory may also depend on the taxonomic group of the plant (Daehler 1998), as plant defense traits such as trichomes, thorns, palatability, and secondary defense chemistry can be conserved within taxonomic groups of plant species (Karban and Baldwin 1997). Multi-species studies with and without herbivores should be considered in this context (Knapp et al. 2008), because most studies that have tested effects of herbivores on plant invasions have focused on monospecific stands or two-species communities (e.g., Lambert and Casagrande 2007; Caño et al. 2009; Sun et al. 2010; Dawson et al. 2014). Confounding effects of taxonomic relatedness can be avoided by analyzing multi-species comparisons using phylogenetically controlled pairs of exotic and native species (Felsenstein 1985; Agrawal et al. 2005; Funk and Vitousek 2007; Funk and Throop 2010; Cushman et al. 2011). This approach may enable the effects of herbivory on an exotic plant to be interpreted independent of general defense responses found in its plant family or genus (Harvey and Purvis 1991; Pyšek and Richardson 2007). However, studies of the effects of herbivory using phylogenetically controlled multi-species plant communities are very rare or possibly nonexistent.

In the work described in the present paper, we examined how aboveground herbivory can change the dominance of native and exotic plants in plant communities during plant community establishment in a riparian ecosystem. Two previous greenhouse studies using plant species from this type of ecosystem showed that successful exotic plant species were less affected by generalist aboveground invertebrate herbivores than native congeners (Engelkes et al. 2008; Macel et al. 2014). In addition, field observations showed that exotics contained fewer herbivorous insects than the related natives (Engelkes et al. 2012). Therefore, we tested the hypothesis that plant communities consisting of exotics and congeneric natives would become dominated by exotic plant species when exposed to herbivores but not when herbivores were excluded.

In order to test our hypothesis, we created experimental field plots with plant communities consisting of six native and six related exotic species (Table 1) that naturally co-occur in the riverine habitats around the Rhine delta in the Netherlands (Tamis et al. 2005; Dirkse et al. 2007). Selecting species from the same ecosystem implied that differences between the native and exotic plant species were more likely to be a consequence of reduced enemy exposure rather than a consequence of differences in the ecology or biology of the species involved (Agrawal et al. 2005; Strauss et al. 2006; Funk and Vitousek 2007). We analyzed effects of herbivory on plant shoot biomass, plant cover, herbivore damage, and shifts in the evenness of plant community composition.

**Table 1:** Characteristics of the plant species used in the field experiment

Species	Status	Origin of exotics	Family	Life	Frequency (log km^2^)		Abbreviation
				History	1975–1988	1988–2000	
*Artemisia biennis*	Exotic	North Asia	Asteraceae	Biennial	2	4	Artbie
*Bidens frondosa*	Exotic	North America	Asteraceae	Annual	8	8	Bidfro
*Bunias orientalis*	Exotic	South-East Europe and West Asia	Brassicaceae	Perennial	3	4	Bunori
*Rorippa austriaca*	Exotic	East Europe	Brassicaceae	Perennial	5	6	Roraus
*Senecio inaequidens*	Exotic	South Africa	Asteraceae	Perennial	6	8	Senina
*Tragopogon dubius*	Exotic	Mid Europe	Asteraceae	Bie/Perennial	2	4	Tradub
*Artemisia vulgaris*	Native		Asteraceae	Perennial	9	9	Artvul
*Bidens tripartita*	Native		Asteraceae	Annual	9	9	Bidtri
*Sinapis arvensis* ^a^	Native		Brassicaceae	Annual	8	9	Sinarv
*Rorippa sylvestris*	Native		Brassicaceae	Perennial	8	9	Rorsyl
*Senecio jacobaea* ^b^	Native		Asteraceae	Bie/Perennial	8	9	Senjac
*Tragopogon pratensis*	Native		Asteraceae	Bie/Perennial	8	8	Trapra

The status of each plant species was either exotic or native (2nd column). All species co-occur in riverine habitats in the Netherlands. Status, origin, and life-history information were based on Tamis et al. (2004, 2005). Frequency is the observed presence, in square kilometers (log value), in the Netherlands during two time periods and is used as an index of abundance and increased regional presence

^a^ *S. arvensis* is an archaeophyte that arrived in the Netherlands before 1500 A.D.

^b^ *Senecio jacobaea* has recently been renamed *Jacobaea vulgaris* (see Pelser et al. 2006)

## Materials and methods

### Study design

We set up a field experiment with five open tents where herbivores were allowed and five control tents where herbivores were excluded. In these tents, mixed communities of six native and six exotic plant species were planted (Table 1; see Fig. S1 of the Electronic supplementary material, ESM, for photos of the experiment). Each exotic plant species was closely related to one of the natives, thus making the comparison phylogenetically controlled. All exotic and native plant species currently co-occur in riverine habitats of the Rhine delta in The Netherlands. The plant communities were established in late spring after germination and initial growth under controlled conditions, and harvested in early fall, when biomass production was slowing down. Some plant species were annuals, whereas most others were biennials or short-lived perennials (Table 1). Therefore, the experiment represents the early establishment phase of the plant communities; most of the annual life cycle, and the establishment phase and early growth of biennials and short-lived perennials.

The field site was situated in a riverine habitat in the nature reserve De Afferdense and Deestse Waarden, situated along the River Waal (the southernmost branch of the Rhine) in between the villages Afferden and Deest, the Netherlands (51°89′N, 5°64′E). The vegetation was removed mechanically and, after soil tillage, ten plots of size 3 × 3 m were created that were separated from each other by 5 m. We established the same plant community in each plot (see Table 1). Three individuals of each plant species were planted, resulting in a total of 3 × (6 + 6) = 36 plants per plot. In every plot, the positions of the individual plants were randomized in a 6 × 6 grid pattern, and the plants were separated from each other by 30 cm. Because of this grid pattern, we expected that there would initially be no competition between the plants; as the growing season proceeded, however, the plants would interfere with each other (Fig. S1b in the ESM). Other naturally emerging plants were kept out by covering the soil with root cloth and by hand-weeding plants that emerged from the planting holes. The experimental communities and natural vegetation were separated by approximately 3 m.

Each of the ten plant communities was enclosed by a 3 × 3 × 2 m (l × w × h) tent of fine nylon mesh (0.5 mm^2^, Rovero Systems BV, Netherlands). The mesh of the tents removed about 20 % of the ambient light (technical datasheet, Rovero Systems BV). We did not include a control for the effect of light inhibition by the tent because that effect could not be separated from the effects of the tent on the microclimate. Five plant communities were exposed to small vertebrates and invertebrates aboveground by opening the entire east side of the tent away from the prevailing wind direction (3 × 2 m opening). This permitted maximum exposure to insects while changing the microclimate and light availability as little as possible. The other five tents were completely closed in order to keep out insects and small vertebrates. Large vertebrates were kept out by placing a fence around the experimental site. We had two rows of five tents in the experimental area. Open and closed tents were alternated both within and between rows in order to minimize possible interference from abiotic gradients in this environment. Distances between neighboring tents were equal both within and between rows. Because of the mesh that was used, all tents were open to natural rainfall. The plant communities in the open tents were exposed to insect herbivory throughout the entire course of the experiment. Rabbits were allowed to enter the tents only for the first 3 weeks. After that, they were kept out of the experimental site by a fence because they would have caused too much damage to the plants. Other small vertebrates such as voles were allowed, but their effects on plants appeared to be minor. Regular checks of the closed tents confirmed that the plant communities in the control treatments were not exposed to aboveground herbivory by insects.

### Plant species selection and seedling growth

The National Standard List of Dutch flora was used to select the plant species that made up our experimental communities (Tamis et al. 2004). Plant species were selected using the same criteria as in previous experiments (Engelkes et al. 2008; Meisner et al. 2011): (1) all exotic and native plant species co-occurred in riverine areas along the River Rhine-Waal (Dirkse et al. 2007); (2) the exotic plants increased in frequency in the second half of the twentieth century (Tamis et al. 2005), to ensure that exotics were recent invaders to the region; and (3) each exotic plant species had a closely related native within the same genus except for *Bunias orientalis*, for which we selected a closely related species within the same family with comparable habitat requirements (*Synapsis arvensis*). A possible drawback of this choice is that *B. orientalis* can be a long-lived perennial, whereas *S. arvensis* is an annual and a so-called archaeophyte that was been introduced before 1500 AD (Table 1).

Seeds were collected from the region where the field site was situated or, in an exceptional case (*Bidens frondosa*), purchased from a specialized seed supplier who collected the seeds locally. Prior to germination, all seeds were surface sterilized using a 1 % hypochlorite solution. Seeds were planted in trays with homogenized sterilized soil (using 25 KGray γ-radiation) that was collected from the same region, and germinated as in Engelkes et al. (2008). Plants were 6 weeks old when transplanted to the field, and individuals that had died due to factors other than herbivory were replanted until the 4th week of the experiment. The field experiment ran from June 1 until September 30, which covers a substantial part of the growing season, including peak plant growth and insect abundance.

### Plant biomass, plant cover, evenness, and herbivore damage

Damage to plants by vertebrate herbivores (rabbits) was determined by visual inspection only on July 31, the peak growth season, and categorized as: 0, no visible damage; 1, <1 %; 2, 1–10 %; 3, 11–50 %; 4, 51–99 %; 5, 100 %. Rabbits were excluded after this initial feeding period. Damage to plants by invertebrate herbivores was also determined by visual inspection of the remaining leaves on July 31. Two additional inspections of the damage caused by invertebrate herbivores were made on August 28 and September 30 (just before the final harvest). In September, feeding damage was exclusively due to aboveground invertebrate herbivores. Damage by invertebrate herbivores was determined as: 0, no visible damage; 1, <1 %; 2, 1–5 %; 3, 5–10 %; 4, 10–50 %; 5, >50 %.

Plant cover was determined as the total surface area covered by individual plant biomass following Cox (1980) at the end of September, after 4 months of growth. Thus, a cover value of 1 would indicate that the plant biomass covers an area of 30 × 30 cm, or 100 % of its pre-assigned square in the community grid. Then, all shoots were clipped to 1 cm above the soil surface and dried at 70 °C for 72 h before determining dry weight. Evenness (the relative contribution of individual species to the total aboveground biomass in each community) was analyzed by calculating Pielou’s* J*, which is calculated as* J* = *H*/ln(*S*) (Pielou 1966); here,* H* is the Shannon–Wiener diversity index and is calculated as *H* = −Σ*p*_*i*_·ln(*p*_*i*_), where *p*_*i*_ is the proportional contribution of the *i*th species to the total aboveground biomass;* S* is the total number of species in a tent.

### Statistical analyses

To account for phylogenetically adjusted species pairs and for non-independence of native and exotic species that were growing in the same plot, we used split-plot mixed-effects ANOVA. Aboveground plant biomass, plant cover, and the proportional contribution of each individual plant to the total aboveground biomass data were analyzed with herbivory, plant status (i.e., native vs exotic origin), genus, and their interactions as fixed effects. Herbivory was included as a whole-plot factor and plant status and genus as crossed subplot factors. Tent identity was included in the model as a random factor to account for non-independence of plants growing in the same tent. We also tested the model herbivory × plant status at the genus level when there was a significant three-way interaction between herbivory, plant status, and genus (see “Results”).The level of visually estimated vertebrate herbivore damage (measured in July) on plants growing in open tents was analyzed using the same split-plot model, but the factor herbivory was excluded from the model. As the level of invertebrate herbivore damage was estimated three times during the experiment (in July, August, and September), a repeated measures mixed-effects model was used to evaluate the changes in invertebrate herbivore damage over time, with status and genus as the between-subject factors and time as the within-subject factor. Tent was also included as a random effect factor in this model. Because the effect of treatment depended on time, we further analyzed the level of invertebrate damage at each time point separately using the same split-plot model as used for vertebrate damage. For the split-plot analyses, we used ANOVA type III SS with the Kenward–Roger approximation to calculate the degrees of freedom in the lmerTest package (Kuznetsova et al. 2015), which makes use of the lme4 package (Bates et al. 2014). One exception was the model for invertebrate herbivore damage, where time was included as the within-subject factor. In this model, the number of degrees of freedom was calculated according to the procedure described in Pinheiro and Bates (2000).

The proportional contribution of each plant species to the total aboveground biomass in a tent was calculated. After that, the proportional contribution of each plant species was ranked from highest to lowest for open and closed tents separately. Testing for similarity of species proportional biomass ranking between control and herbivory treatments was done with a Kendall′s* W* for concordance test.

Total aboveground community biomass, Shannon community evenness, and community Pielou’s* J* in open tents were compared with those in closed tents using a *t*-test (*n* = 5 per treatment). The effect of herbivory on plant community composition (the proportional biomass of each plant species with respect to the total community biomass) was tested using a principal components analysis (PCA) and redundancy analysis (RDA) (999 unrestricted permutations, CANOCO, v.5.03; Šmilauer and Lepš 2014). As there were three plant individuals of each species growing within a single tent, the proportional biomass of each plant was averaged per tent before the analyses. This analysis showed the same effects as when the three individuals were not averaged (data not shown), but resulted in a clearer visual presentation.

To test if the measured damage (from vertebrates in July and invertebrates in July, August, and September) could explain the relative difference in biomass between the control and herbivory treatments at the end of September, the difference in shoot biomass between the control and herbivory treatments for each species was calculated as ln (shoot biomass without herbivory/shoot biomass with herbivory) and correlated to the corresponding species-specific damage levels by Spearman’s rank correlation. We used Spearman’s rho, which explicitly accounts for ties (Zar 1984).

Prior to analyses, biomass and cover data were log transformed to fulfill assumptions of normality and homogeneity of variances. Unless indicated otherwise, data were analyzed with R version 3.0.1 (R Development Core Team 2014).

## Results

**Fig. 1:**
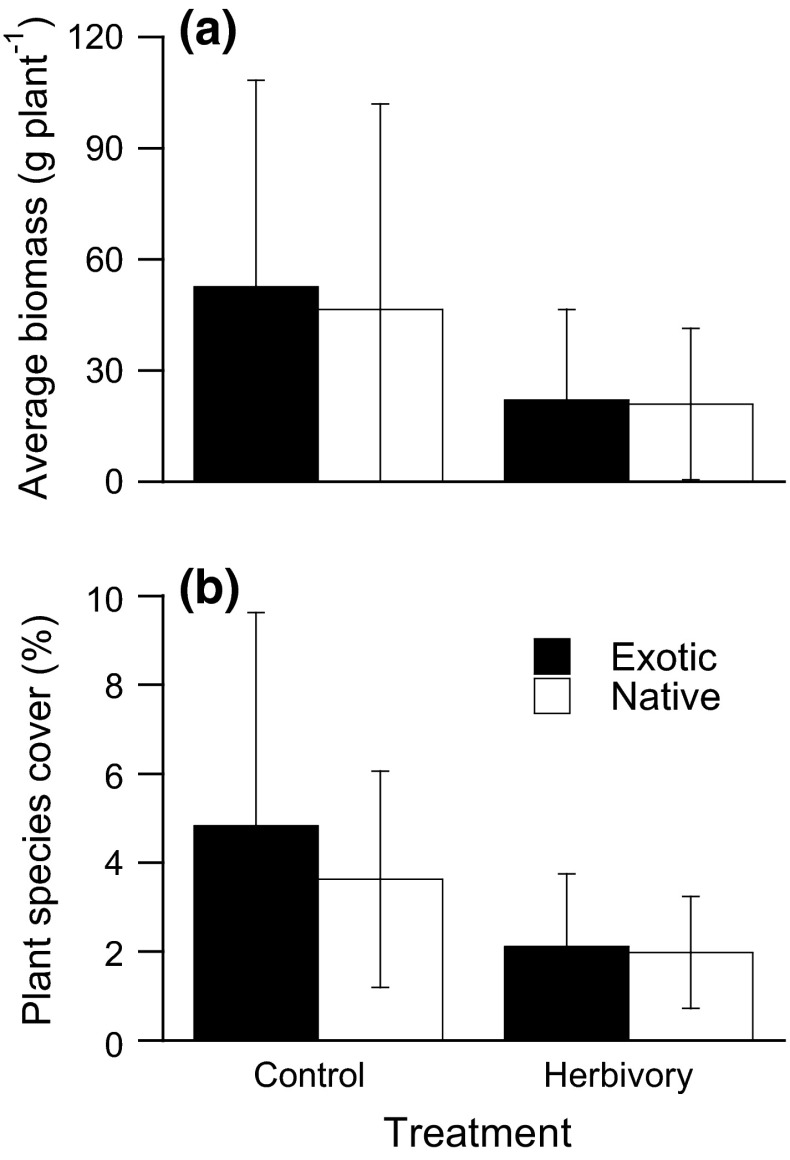
Effects of treatment on **a** average aboveground biomass and **b** average plant cover (%) for exotic and native plant species. Values are mean ± SD; means were determined by averaging species values within a tent and then for each treatment

Aboveground biomass of exotic plant species did not differ from that of native plant species in communities without herbivory or in communities with herbivory (herbivory × status: Table 2; Fig. 1a). However, the effect of herbivory on aboveground biomass depended on genus and status (herbivory × status × genus; Table 2). The origin of this three-way interaction can be understood by inspecting the individual species responses (Fig. S2 in the ESM): aboveground biomass was lower for both exotic and native species when exposed to herbivory in the cases of* Bidens* and* Artemisia*, but did not differ between open and control tents for either exotic or native species in the cases of *Rorippa* and *Tragopogon*; in the cases of *Senecio* and *Bunias*, native and exotic species responded different to herbivory (Fig. S2 in ESM). Overall, plant communities exposed to herbivory had, on average, less biomass per plant than communities without herbivory, and herbivory reduced the biomasses of both exotic and native plant species (Table 2; Fig. 1a). Exposure to aboveground herbivores also reduced average plant community cover (Table 2; Fig. 1b). Plant communities exposed to aboveground herbivores were not dominated more by exotic plant species than communities without herbivory (Table 2; Fig. 1b), but the cover of exotic or native species depended on genus (herbivory × status × genus; Table 2; Fig. S3 in the ESM).

**Table 2:** Effects of herbivory, plant status (native vs exotic), and genus on aboveground plant biomass, plant cover, and the proportional contribution of each species to community biomass

	*df*	Aboveground biomass (*N* = 326)	Cover (*N* = 325)	Proportional biomass (*N* = 333)
Herbivory (H)	1,8	41.19***	40.38***	0.046
Status (S)	1,N	1.31	0.052	0.22
Genus (G)	5,N	6.99***	8.14***	17.13***
H × S	1,N	0.34	0.55	0.19
H × G	5,N	24.64***	28.58***	18.31***
S × G	5,N	4.19**	4.20**	7.44***
H × S × G	5,N	5.24***	5.19***	1.44

Degrees of freedom (*df*) and *F* values are shown for split-plot mixed-effects ANOVA. *Asterisks* indicate significant effects: *** *P* < 0.001; ** *P* < 0.01

**Fig. 2:**
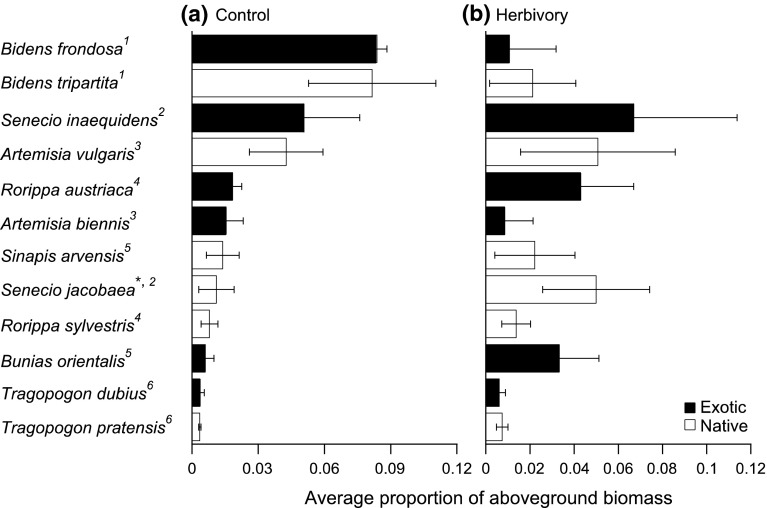
Proportional rank abundance plots of the average (±SD) proportions of aboveground biomass of species in the plant communities in **a** control (closed tents) and **b** herbivory (open tents) treatments.* Numbers after plant names* indicate species pairs: ^1^ *Bidens*, ^2^ *Senecio*, ^3^ *Artemisia*, ^4^ *Rorippa*, ^5^ *Sinapis*/*Bunias*, ^6^ *Tragopogon*. * synonym is *Jacobaea vulgaris* (see Table 1)

The effects of herbivory and plant status on the proportional biomass of a species depended on genus (Table 2; Fig. 2). In contrast, herbivory altered the rank of proportional biomass of a species within the community, although the shapes of the rank abundance plots with and without herbivory were not significantly different from each other (Kendall’s* W* of concordance, *n* = 12, *W* = 0.303, *P* = 0.19; Fig. 2). Some plant species were extremely sensitive to herbivory. For example, the exotic and native *Bidens* species produced 30 and 33 % of the aboveground community biomass, respectively, in the absence of herbivory, whereas these percentages dropped to less than 4 and 7 %, respectively, when herbivores were allowed (Fig. 2). Other plant species benefited from herbivory, at least proportionally. For example, the exotic *Senecio inaequidens* produced more than 15 % of the aboveground biomass in communities without herbivory, and 20 % when herbivores were allowed (Fig. 2). The natives *Artemisia vulgaris* and *Senecio jacobaea* as well as the exotics *Rorippa austriaca* and *B. orientalis* were all ranked relatively high in the communities with herbivory (Fig. 2).

**Fig. 3:**
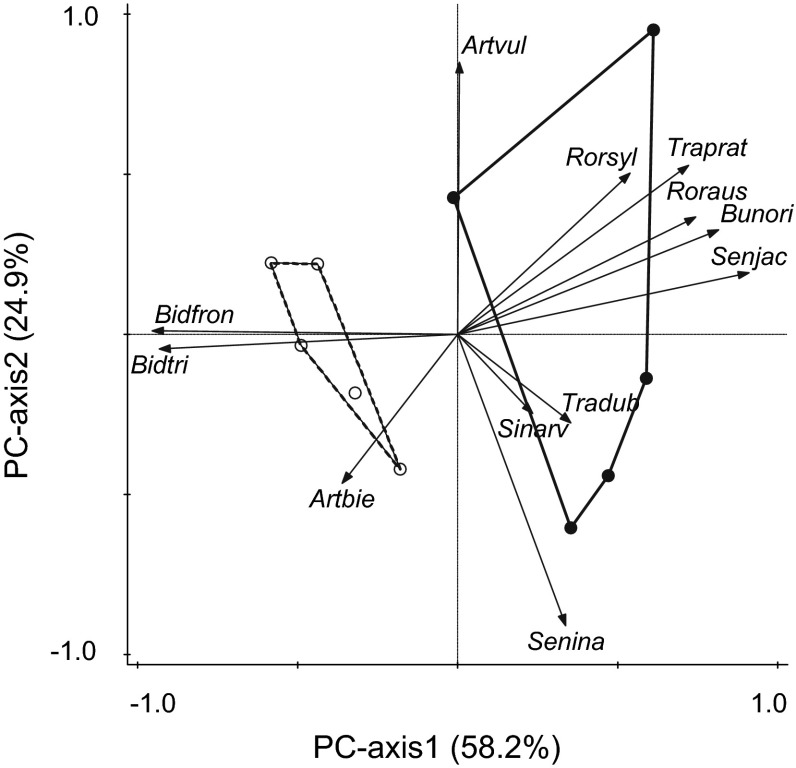
PCA ordination of proportion of aboveground biomass of each species in control (*open* *dots*, *dashed* *line*) and herbivory (*closed* *dots*, *solid* *line*) treatments for exotic and native plant species. Each* number in parentheses* indicates the percentage of the variation explained by the axis. For full species names, see Table 1

Total community biomass was significantly lower in open tents compared to control tents (Table 3). There was no difference in total community Shannon index and Pielou’s* J* between open and control tents (Table 3). Herbivory explained 48.2 % of the total variation in the composition of the plant community (RDA, *F* = 7.5, *P* = 0.01; Fig. 3). Plant communities exposed to herbivory had higher dissimilarity in aboveground biomass distribution among species than plant communities without herbivory (Fig. 3).

**Table 3:** Total community biomass, Shannon index, and Pielou’s* J* in control and herbivory treatments (values are mean ± SE; *n* = 5)

	Control	Herbivory	*df*	*t*	*P*
Total biomass	1730 ± 195	783 ± 82	5.38	4.47	0.006
Shannon index	0.91 ± 0.10	0.97 ± 0.04	4.53	0.81	0.46
Pielou’s* J*	0.37 ± 0.04	0.41 ± 0.02	4.33	−0.42	0.69

Results of *t*-tests are shown for each comparison

**Fig. 4:**
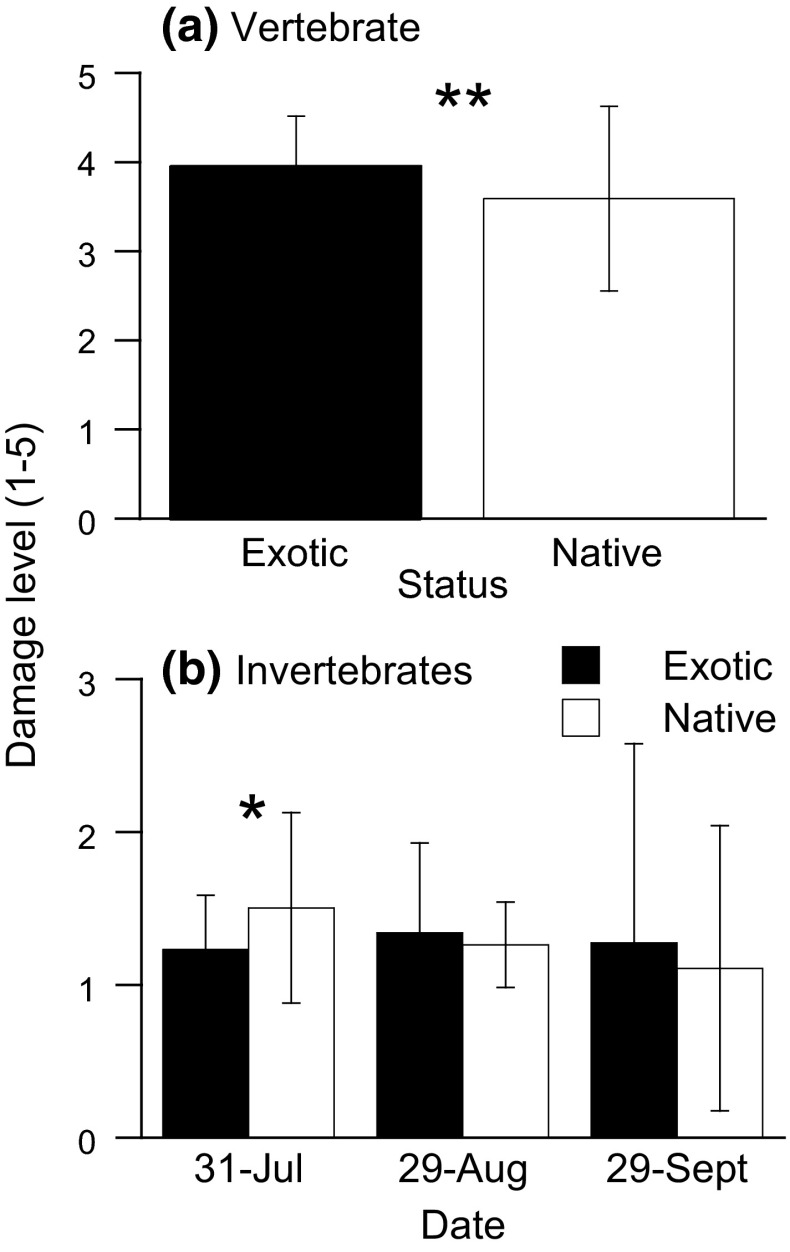
Damage level by **a** vertebrate and **b** invertebrate herbivores on exotic and native plants in open tents where herbivores were allowed. Values are means  ± SD; categorization in **a** is:* 0* is no visible damage;* 1* < 1 %;* 2* is 1–10 %;* 3* is 11–50 %;* 4* is 51–99 %;* 5* is 100 %, and that in **b** is:* 0* is no visible damage;* 1* < 1 %;* 2* is 1–5 %;* 3* is 5–10 %;* 4* is 10–50 %;* 5* is > 50 %. Damage by invertebrates was measured three times over the experiment (in July, August, and September). *Asterisks* indicate significant differences between exotics and natives. *P < 0.05 and **P < 0.01

Overall, exotic plant species were damaged more than natives by vertebrate herbivores (mostly rabbits) in July (*F*_1,163_ = 9.53, *P* = 0.002; Fig. 4a). However, the difference in the levels of vertebrate damage on natives and exotics also depended on plant genus (genus × status interaction: *F*_5,163_ = 21.22, *P* < 0.001; Fig. S4 in the ESM). The exotic *B. orientalis* was less damaged by vertebrate herbivores than the native congener, whereas the exotic *S. inaequidens* was damaged more than the native congener (Fig. S4 in the ESM).

We did not identify all aboveground invertebrate herbivores that were damaging plants, but a number of herbivorous insects were observed feeding on the plant tissues in the open cages: *Longitarsusjacobaeae*, *Pierisrapae*, and *Colaphussophiae*, among others. The difference in the levels of invertebrate damage on native and exotic plants depended on plant genus (status × genus interaction: *F*_5,109_ = 16.47, *P* < 0.001) and varied over time when it was measured (Fig. 4b). The level of invertebrate damage was significantly higher on native than on exotic plant species in July only (*F*_1,108_ = 6.28, *P* = 0.014; Fig. 4b). Throughout the growing season, the difference in the levels of invertebrate damage on native and exotic plants depended on genus (July: *F*_5,108_ = 6.30, *P* < 0.001; August: *F*_5,109_ = 4.01, *P* = 0.002; September: *F*_5,106_ = 20.63, *P* < 0.001; Fig. S5 in the ESM).

The level of visually assessed feeding damage by vertebrate herbivores (measured in July only) was positively correlated to the ratio of aboveground biomass in open to that in closed cages (Spearman’s rank correlation: *ρ* = 0.92, *n* = 12, *P* < 0.001). There was no significant correlation between the level of visually assessed feeding damage by invertebrate herbivores and the ratio of aboveground biomass in open to closed cages throughout the season (July: *ρ* = −0.28, *n* = 12, *P* = 0.37; August: *ρ* = −0.53, *n* = 12, *P* = 0.08; September: *ρ* = −0.28, *n* = 12, *P* = 0.38).

## Discussion

We studied the effects of aboveground herbivory on phylogenetically controlled multi-species communities of exotic and native plant species during their first growing season in the field. In contrast to our hypothesis, herbivory did not enhance the overall dominance of exotic plant species. Instead, plant communities were co-dominated by both native and exotic plant species. Herbivory reorganized the rank order of native and exotic species in the plant communities. Some native plant species, such as *S. jacobaea (*syn. *Jacobaea vulgaris*) performed substantially better under herbivory than some of the exotic plant species. The response of this species is well in line with expectations, as *S. jacobaea* is known to be a poor competitor with other plant species (McEvoy et al. 1993), whereas it is known to be toxic to herbivores aside from some specialized invertebrates (Macel 2011). The close exotic relative *S. inaequidens* is also toxic (Engelkes et al. 2008), but appeared more competitive than *S. jacobaea* when herbivores were excluded.

Studies showing that invasive exotic plant species in general have fewer enemies (Mitchell and Power 2003), even when compared to congeneric natives (Engelkes et al. 2012), and that they are less damaged by aboveground herbivores than phylogenetically related natives (Engelkes et al. 2008; Funk and Throop 2010) suggest that invaded plant communities that are exposed to herbivores will become dominated by exotics. However, our results do not confirm this expectation. It has been argued that focusing on the number of enemies present might not necessarily be a good proxy for effects on biomass production (van Kleunen and Fischer 2009). Our results suggest that the abovementioned proxies may not necessarily be good predictors of the outcome of plant community interactions when the communities are exposed to natural herbivory in the field.

Comparisons among studies may be complicated by the presence of different environmental contexts. For example, in another study, *B. frondosa* was not affected by invertebrate herbivores when grown in pots in the absence of competitors (Dawson et al. 2014). In our community approach, however, this plant species lost dominance in the community exposed to herbivory; this was probably due to grazing intolerance to vertebrate herbivores, which prevented regrowth after excluding the rabbits. This may be an example of how plant–herbivore relationships can depend on environmental context. A number of the plant species that we used in the field experiment have been exposed individually to herbivory under controlled conditions. In those studies, aboveground generalist herbivorous insects were, on average, eating more from native than from phylogenetically related exotic plant species (Engelkes et al. 2008; Fortuna et al. 2012, 2013; Macel et al. 2014). The current field study shows that even those results are not generally indicative of the performance of exotics versus the performance of natives in a plant community context in the field.

Contrasting results between our previous greenhouse experiments (Engelkes et al. 2008; Macel et al. 2014), field observations (Engelkes et al. 2012), and the current plant community experiment might be explained by the relatively short duration of our field experiment, which may have underestimated effects on seed production and plant regrowth after winter. These factors can influence long-term community dynamics. Another possible explanation might be that greenhouse experiments on feeding by single insect species and field surveys on insect abundance may not be indicative of how plants actually perform under natural herbivory in the field. For example, we found that, on average, exotic plant species showed more damage by vertebrate herbivores (at least in July) than the natives. The fact that this damage did not result in a greater loss of biomass could indicate a higher tolerance to herbivory. Tolerance to herbivory has been suggested as an alternative explanation for the success of invasive species (Augustine and McNaughton 1998), but there is no general evidence that invasive exotic plant species are more tolerant to herbivory than native plant species (Chun et al. 2010).

Relatively few studies have examined the effects of herbivory on exotic plant species in plant communities in field experiments. These studies have provided mixed evidence regarding the role of herbivores in promoting exotic plant invasions. For example, grazing by larger vertebrate herbivores such as cows and sheep did not accelerate exotic plant spread in a large grassland experiment (Stohlgren et al. 1999). In another study, grazing by vertebrate herbivores promoted the abundance of exotic species that suffered less from grazing than natives (HilleRisLambers et al. 2010). It is possible that the effects of herbivorous insects depend on the phylogenetic relationship between native and exotic plant species (Ashton and Lerdau 2008). This might be due to greater similarity in resistance traits to herbivores between congeneric plants than between distantly related species (Agrawal 2007). For example, in our study, both the exotic and native *Bidens* species were found to be highly sensitive to aboveground herbivory. As such, this species pair may have driven the patterns for average biomass, cover, and total biomass. Alternatively, it is also possible that herbivores on native plant species shift more easily to exotic congenerics than to unrelated species (Strauss et al. 2006), resulting in similar levels of damage to exotics and natives in the field observations (Dostál et al. 2013).

Exotic plant success has also been hypothesized to depend on the identity of the dominant native plant species in the community (Emery 2007; Emery and Gross 2007). Our results show that the presence or absence of herbivory influences which of the plant species dominate. Only for the* Bunias*–*Sinapis* pair did the species respond in line with our proposed hypothesis, as *B. orientalis* performed better under herbivory than in the control plots (Fig. S2 in the ESM). In all of the other pairs, the exotic species did not respond in line with our hypothesis—they performed worse or were not affected by exposure to herbivory. Effects of aboveground herbivory on invasive exotic plant species generally tend to be highly variable between studies. Some studies have even shown that exotic plants were preferred by herbivores over native plants (Parker and Hay 2005; Parker et al. 2006), whereas exotics and natives responded similarly to herbivores in other studies (Dawson et al. 2014). These examples and our own results suggest that effects of herbivores on introduced exotic plant species need to be considered in their natural context of intra- and interspecific plant interactions.

We conclude that herbivory does not promote the overall supremacy of exotics during the establishment of mixed plant communities of exotic species and phylogenetically related natives. Our conclusion is based on a relatively short-term field experiment that did not include regeneration of plant communities after one or several winter periods. Moreover, it may be that such phylogenetically balanced communities do not occur naturally in the field, even when all plant species are inhabitants of the same riverine ecosystem. But what our experiment does suggest is that effects of aboveground herbivory on exotic versus native plant species may strongly depend on their degree of relatedness, and thus their defense strategies. Therefore, we propose that more experiments which adopt a plant community approach and include phylogenetic relatedness between exotics and natives are needed to test the current assumptions regarding the consequences of less enemy exposure of exotic plant species in their novel ranges for plant invasiveness.

## Electronic supplementary material

Supplementary material 1 (DOCX 1075 kb)

## References

[CR1] Agrawal AA (2007). Macroevolution of plant defense strategies. Trends Ecol Evol.

[CR2] Agrawal AA, Kotanen PM, Mitchell CE, Power AG, Godsoe W, Klironomos J (2005). Enemy release? An experiment with congeneric plant pairs and diverse above- and belowground enemies. Ecology.

[CR3] Ashton IW, Lerdau MT (2008). Tolerance to herbivory, and not resistance, may explain differential success of invasive, naturalized, and native North American temperate vines. Divers Distrib.

[CR4] Augustine DJ, McNaughton SJ (1998). Ungulate effects on the functional species composition of plant communities: herbivore selectivity and plant tolerance. J Wildl Manag.

[CR5] Bates D, Maechler M, Bolker B, Walker S (2014) lme4: linear mixed-effects models using Eigen and S4. R package version 1.1-7. http://CRAN.R-project.org/package=lme4%3E. Accessed 16 July 2015

[CR6] Caño L, Escarré J, Vrieling K, Sans F (2009). Palatability to a generalist herbivore, defence and growth of invasive and native *Senecio* species: testing the evolution of increased competitive ability hypothesis. Oecologia.

[CR7] Catford JA, Jansson R, Nilsson C (2009). Reducing redundancy in invasion ecology by integrating hypotheses into a single theoretical framework. Divers Distrib.

[CR8] Chun YJ, van Kleunen M, Dawson W (2010). The role of enemy release, tolerance and resistance in plant invasions: linking damage to performance. Ecol Lett.

[CR9] Colautti RI, Ricciardi A, Grigorovich IA, MacIsaac HJ (2004). Is invasion success explained by the enemy release hypothesis?. Ecol Lett.

[CR10] Cox GW (1980). Laboratory manual of general ecology.

[CR11] Cushman JH, Lortie CJ, Christian CE (2011). Native herbivores and plant facilitation mediate the performance and distribution of an invasive exotic grass. J Ecol.

[CR12] Daehler CC (1998). The taxonomic distribution of invasive angiosperm plants: ecological insights and comparison to agricultural weeds. Biol Conserv.

[CR13] Dawson W, Bottini A, Fischer M, van Kleunen M, Knop E (2014). Little evidence for release from herbivores as a driver of plant invasiveness from a multi-species herbivore-removal experiment. Oikos.

[CR14] Dirkse GM, Hochstenbach SMH, Reijerse AI (2007). Flora van Nijmegen en Kleef 1800-2006/Flora von Nimwegen und Kleve 1800-2006.

[CR15] Dostál P, Allan E, Dawson W, van Kleunen M, Bartish I, Fischer M (2013). Enemy damage of exotic plant species is similar to that of natives and increases with productivity. J Ecol.

[CR16] Emery SM (2007). Limiting similarity between invaders and dominant species in herbaceous plant communities?. J Ecol.

[CR17] Emery SM, Gross KL (2007). Dominant species identity, not community evenness, regulates invasion in experimental grassland plant communities. Ecology.

[CR18] Engelkes T, Morriën E, Verhoeven KJF, Bezemer TM, Biere A, Harvey JA, McIntyre LM, Tamis WLM, van der Putten WH (2008). Successful range-expanding plants experience less above-ground and below-ground enemy impact. Nature.

[CR19] Engelkes T, Wouters B, Bezemer TM, Harvey JA, van der Putten WH (2012). Contrasting patterns of herbivore and predator pressure on invasive and native plants. Basic Appl Ecol.

[CR20] Felsenstein J (1985). Phylogenies and the comparative method. Am Nat.

[CR21] Fortuna TM, Vet LEM, Harvey JA (2012). Effects of an invasive plant on the performance of two parasitoids with different host exploitation strategies. Biol Control.

[CR22] Fortuna T, Woelke J, Hordijk C, Jansen J, van Dam N, Vet LM, Harvey J (2013) A tritrophic approach to the preference–performance hypothesis involving an exotic and a native plant. Biol Invasions 15(11):2387–2401. doi:10.1007/s10530-013-0459-2

[CR23] Funk JL, Throop HL (2010). Enemy release and plant invasion: patterns of defensive traits and leaf damage in Hawaii. Oecologia.

[CR24] Funk JL, Vitousek PM (2007). Resource-use efficiency and plant invasion in low-resource systems. Nature.

[CR25] Gonzales EK, Arcese P (2008). Herbivory more limiting than competition on early and established native plants in an invaded meadow. Ecology.

[CR26] Harvey PH, Purvis A (1991). Comparative methods for explaining adaptations. Nature.

[CR27] HilleRisLambers J, Yelenik SG, Colman BP, Levine JM (2010). California annual grass invaders: the drivers or passengers of change?. J Ecol.

[CR28] Holmgren M, Aviles R, Sierralta L, Segura AM, Fuentes ER (2000). Why have European herbs so successfully invaded the Chilean matorral? Effects of herbivory, soil nutrients, and fire. J Arid Environ.

[CR29] Jobin A, Schaffner U, Nentwig W (1996) The structure of the phytophagous insect fauna on the introduced weed* Solidago altissima* in Switzerland. Entomol Exp Appl 79(1):33–42. doi:10.1111/j.1570-7458.1996.tb00806.x

[CR30] Karban R, Baldwin IT (1997). Induced responses to herbivory.

[CR31] Keane RM, Crawley MJ (2002). Exotic plant invasions and the enemy release hypothesis. Trends Ecol Evol.

[CR32] Knapp LB, Fownes JH, Harrington RA (2008). Variable effects of large mammal herbivory on three non-native versus three native woody plants. For Ecol Manag.

[CR33] Kuznetsova A, Brockhoff PB, Christensen RHB (2015) lmerTest: Tests in Linear Mixed Effects Models. R package version 2.0-25. http://CRAN.R-project.org/package=lmerTest. Accessed 16 July 2015

[CR34] Lambert AM, Casagrande RA (2007) Susceptibility of native and non-native common reed to the non-native mealy plum aphid (Homoptera: Aphididae) in North America. Environ Entomol 36(2):451–457. doi:10.1603/0046-225X(2007)36[451:SONANC]2.0.CO;2 10.1603/0046-225x(2007)36[451:sonanc]2.0.co;2 17445381

[CR35] Levine JM, Vila M, D’Antonio CM, Dukes JS, Grigulis K, Lavorel S (2003). Mechanisms underlying the impacts of exotic plant invasions. Proc R Soc Lond B Biol Sci.

[CR36] Liu H, Stiling P (2006). Testing the enemy release hypothesis: a review and meta-analysis. Biol Invasions.

[CR37] Lodge DM (1993). Biological invasions—lessons for ecology. Trends Ecol Evol.

[CR38] Macel M (2011). Attract and deter: a dual role for pyrrolizidine alkaloids in plant–insect interactions. Phytochem Rev.

[CR39] Macel M, de Vos RCH, Jansen JJ, van der Putten WH, van Dam NM (2014). Novel chemistry of invasive plants: exotic species have more unique metabolomic profiles than native congeners. Ecol Evol.

[CR40] Mack RN, Simberloff D, Lonsdale WM, Evans H, Clout M, Bazzaz FA (2000) Biotic invasions: causes, epidemiology, global consequences, and control. Ecol Appl 10(3):689–710. doi:10.1890/1051-0761(2000)010[0689:BICEGC]2.0.CO;2

[CR101] McEvoy PB, Rudd NT, Cox CS, Huso M (1993). Disturbance, competition, and herbivory effects on ragwort Senecio jacobaea populations. Ecological Monographs.

[CR41] Meisner A, de Boer W, Verhoeven KJF, Boschker HTS, van der Putten WH (2011). Comparison of nutrient acquisition in exotic plant species and congeneric natives. J Ecol.

[CR42] Memmott J, Fowler SV, Paynter Q, Sheppard AW, Syrett P (2000). The invertebrate fauna on broom, *Cytisus scoparius*, in two native and two exotic habitats. Acta Oecol Int J Ecol.

[CR43] Mitchell CE, Power AG (2003). Release of invasive plants from fungal and viral pathogens. Nature.

[CR44] Newingham BA, Callaway RM (2006). Shoot herbivory on the invasive plant, *Centaurea maculosa*, does not reduce its competitive effects on conspecifics and natives. Oikos.

[CR45] Parker IM, Gilbert GS (2007). When there is no escape: the effects of natural enemies on native, invasive, and noninvasive plants. Ecology.

[CR46] Parker JD, Hay ME (2005). Biotic resistance to plant invasions? Native herbivores prefer non-native plants. Ecol Lett.

[CR47] Parker JD, Burkepile DE, Hay ME (2006). Opposing effects of native and exotic herbivores on plant invasions. Science.

[CR48] Pelser PB, Veldkamp JF, Van der Meijden R (2006). New combinations in *Jacobaea* Mill. (Asteraceae: Senecioneae). Compos Newsl.

[CR49] Pielou EC (1966). The measurement of diversity in different types of biological collections. J Theor Biol.

[CR50] Pinheiro JC, Bates DM (2000). Mixed-effects models in S and S-Plus.

[CR51] Prieur-Richard A-H, Lavorel S, Linhart Y, Dos Santos A (2002). Plant diversity, herbivory and resistance of a plant community to invasion in Mediterranean annual communities. Oecologia.

[CR52] Pyšek P, Richardson DM (2007) Traits associated with invasiveness in Alien plants: where do we stand? In: Nentwig W (ed) Biological invasions. Ecological studies, vol 193. Springer, Heidelberg, pp 97–125. doi:10.1007/978-3-540-36920-2_7

[CR100] R Development Core Team (2014) R: A Language and Environment for Statistical Computing. R Foundation for Statistical Computing, Vienna, Austria

[CR53] Richardson DM, Pyšek P, Rejmanek M, Barbour MG, Panetta FD, West CJ (2000). Naturalization and invasion of alien plants: concepts and definitions. Divers Distrib.

[CR54] Šmilauer P, Lepš J (2014). Multivariate analysis of ecological data using CANOCO 5.

[CR55] Stohlgren TJ, Schell LD, Vanden Heuvel B (1999). How grazing and soil quality affect native and exotic plant diversity in rocky mountain grasslands. Ecol Appl.

[CR56] Strauss SY, Webb CO, Salamin N (2006). Exotic taxa less related to native species are more invasive. Proc Natl Acad Sci USA.

[CR57] Sun Y, Ding J, Frye MJ (2010). Effects of resource availability on tolerance of herbivory in the invasive *Alternanthera philoxeroides* and the native *Alternanthera sessilis*. Weed Res.

[CR58] Tamis WLM, Rvd Meijden, Runhaar J, Bekker RM, Ozinga WA, Odé B, Hoste I (2004). Standaardlijst van de Nederlandse flora 2003. Gorteria.

[CR59] Tamis WLM, Van’t Zelfde M, Van der Meijden R, De Haes HAU (2005). Changes in vascular plant biodiversity in the Netherlands in the 20th century explained by their climatic and other environmental characteristics. Clim Chang.

[CR60] Theoharides KA, Dukes JS (2007). Plant invasion across space and time: factors affecting nonindigenous species success during four stages of invasion. New Phytol.

[CR61] Torchin ME, Lafferty KD, Dobson AP, McKenzie VJ, Kuris AM (2003). Introduced species and their missing parasites. Nature.

[CR62] Van Kleunen M, Fischer M (2009). Release from foliar and floral fungal pathogen species does not explain the geographic spread of naturalized North American plants in Europe. J Ecol.

[CR63] Wolfe LM, Elzinga JA, Biere A (2004). Increased susceptibility to enemies following introduction in the invasive plant *Silene latifolia*. Ecol Lett.

[CR64] Zar JH (1984) Biostatistical analysis. Prentice-Hall, Upper Saddle River

