# Supplementary material for: Herbivory and dominance shifts among exotic and congeneric native plant species during plant community establishment
Source: Oecologia. 2015 Oct 19;180:507–17. doi: 10.1007/s00442-015-3472-6 (PMC4723625; doi:10.1007/s00442-015-3472-6)
Supplement: Supplementary file 1 — Supplementary material 1 (DOCX 1075 kb) [file 442_2015_3472_MOESM1_ESM.docx]

Electronic Supplementary Material associated with:

**Herbivory and dominance shifts among exotic and congeneric native plant species during plant community establishment**

Tim Engelkes ^1+^, Annelein Meisner ^1+§^, Elly Morriën ^1^, Olga Kostenko^1^, Wim H. Van der Putten ^1,2*^, Mirka Macel ^1#^

^1^Department of Terrestrial Ecology

Netherlands Institute of Ecology (NIOO-KNAW)

P.O. Box 50, 6700 AB Wageningen, The Netherlands

^2^Laboratory of Nematology

Wageningen University and Research Centre

P.O. Box 8123, 6700 ES Wageningen, The Netherlands

§ Present addresses: Microbial Ecology Group, Department of Biology, Lund University, Ecology building, SE-223 62 Lund, Sweden; Sections of Microbiology and Terrestrial Ecology, Department of Biology, University of Copenhagen, Universitetsparken 15, bygning 1, 2100 København Ø, Denmark

# Present address: University of Tuebingen, Department of Plant Ecology, Auf der Morgenstelle 5, 72076 Tuebingen, Germany

^+^ Both authors have contributed equally

*Corresponding author: [w.vanderputten@nioo.knaw.nl](mailto:w.vanderputten@nioo.knaw.nl) Department of Terrestrial Ecology, Netherlands Institute of Ecology (NIOO-KNAW), P.O. Box 50, 6700 AB Wageningen, The Netherlands. Phone +31 317473400

**
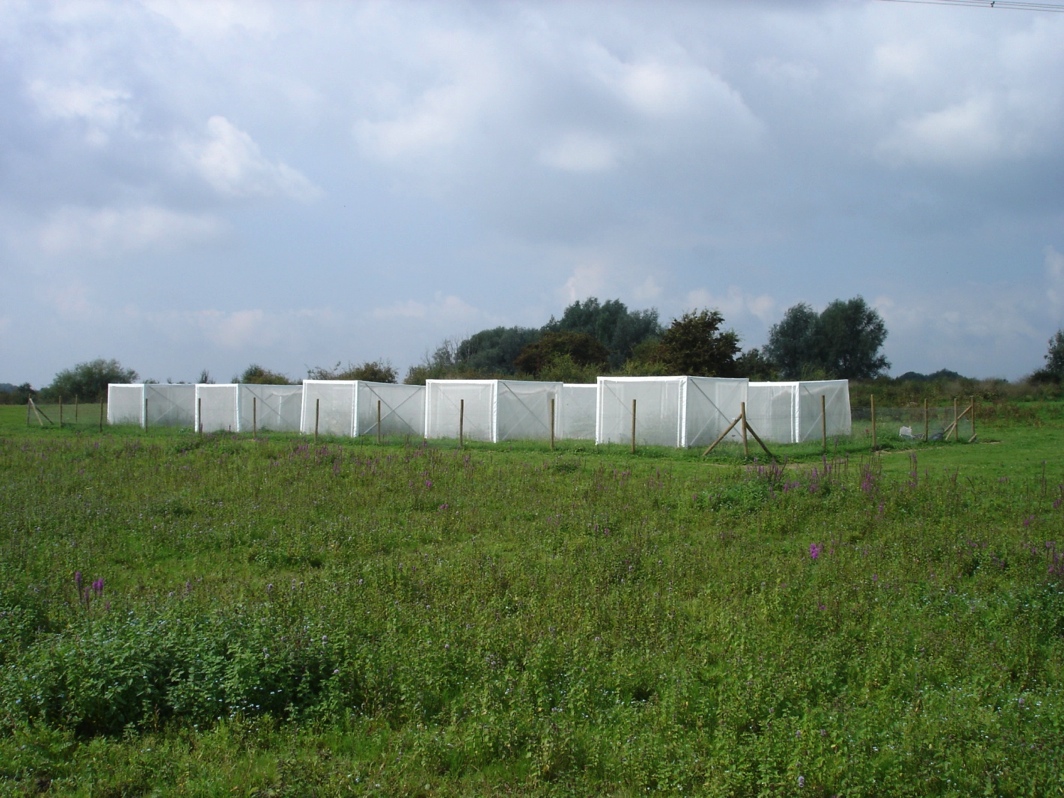
**

**a**


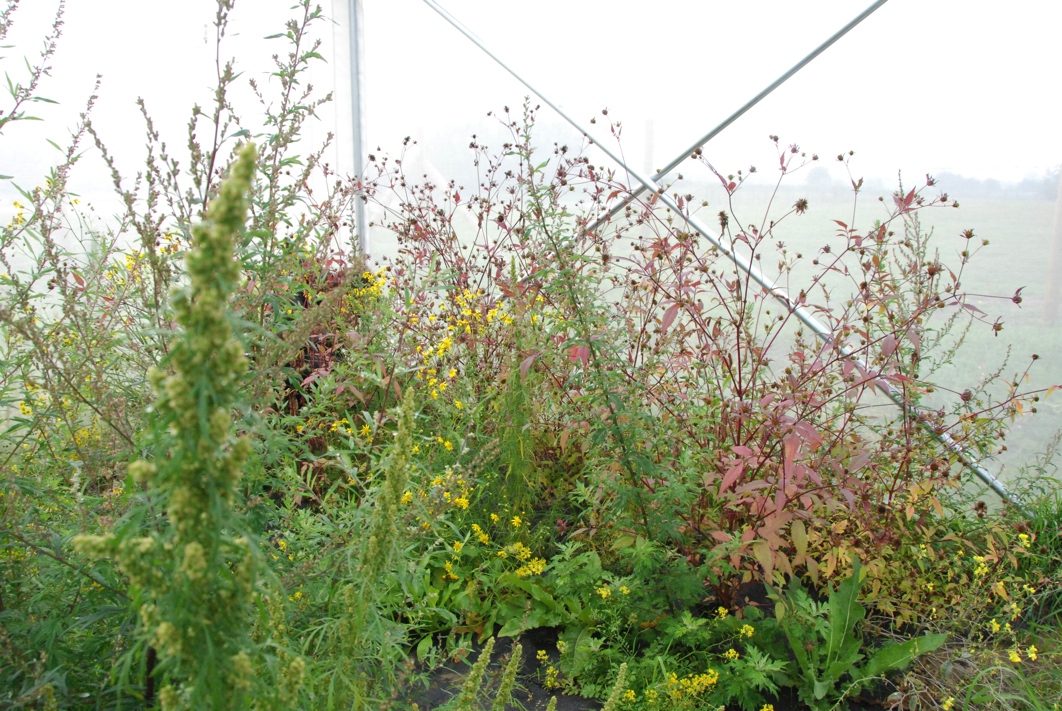


**b**

**Fig. S1**. a) Overview of the experiment in the Afferdense and Deestse Waarden from the wind-ward side. At the rear, tents exposed to herbivory were open. b) Plant community within a control tent.

**Fig. S2** Effect of herbivory on aboveground biomass of native and exotic plant species growing in mixed communities Means ± SD are shown for each genus (note difference in scale of the y-axis). Presented averages were obtained by averaging per species within tent before the community mean was calculated. The outcomes of the split-plot model Status x Herbivory is shown for each genus with **P < 0.01: ***P<0.001.

**Fig. S3** Effect of herbivory on cover of native and exotic plant species growing in mixed communities Means ± SD are shown for each genus (note difference in scale of the y-axis). Presented averages were obtained by averaging per species within tent before the community mean was calculated. The outcomes of the model Status x Herbivory is shown for each genus with ** P < 0.01: ***P<0.001.

**Fig. S4** Damage by vertebrate herbivores to native and exotic plant species growing in mixed communities. Means ± SD are shown. ***P<0.001 for pairwise comparison between exotic and native species within Genus-pair.

**
Fig. S5** Damage by invertebrate herbivores to native (white bars) and exotic (black bars) plant species growing in mixed communities throughout the growing season. Means ± SD are shown for each time point (note difference in scale of the y-axis). **P<0.01 and ***P<0.001 for pairwise comparison between exotic and native species within Genus-pair.

*
